# Prolonged menstruation and increased menstrual blood with generalized δ electroencephalogram power: A case report

**DOI:** 10.3892/etm.2014.1473

**Published:** 2014-01-03

**Authors:** FENGHUA PENG, LIANPING ZHANG

**Affiliations:** Department of Urology, Second Xiangya Hospital, Central South University, Changsha, Hunan 410011, P.R. China

**Keywords:** prolonged menstruation, estradiol, δ frequency, electroencephalogram

## Abstract

Estradiol changes associated with the menstrual cycle have a great impact on brain activation. δ frequency mainly appears during normal sleep status or brain injury diseases, including encephalitis and mental confusion. The current case report presents a 51-year-old female with prolonged menstruation and increased menstrual blood volume whose electroencephalogram (EEG) recording demonstrated a rare generalized 3 Hz δ frequency band in the waking status. The patient had been suffering from heart palpitations and dizziness for 6 months and was receiving treatment in the Department of Neurology (Second Xiangya Hospital). The individual had been experiencing prolonged menstruation and increased menstrual blood volume for 6 years. Gynecologial examination revealed secondary anemia and hysteromyoma. Hemoglobin levels were decreased to 69 g/l. Physical and neurological examinations, and computed tomography results appeared normal. The EEG recording indicated a generalized 3 Hz δ frequency band with 30–80 μV power and a long-range δ frequency band when the patient was hyperventilating. The prolonged menstruation and increased menstrual blood volume may have induced the generalized δ frequency without brain injury. To the best of our knowledge, this is the first formal case report of prolonged menstruation and increased menstrual blood volume with the abnormality of δ EEG power.

## Introduction

Previous studies have indicated that there is a relationship between ovarian hormones and central nervous system function. An overall increase in brain activation with enhanced estradiol levels is observed during the menstrual cycle (1,2). Progesterone, which is synthesized in the ovary, is the most important progestin in humans and has cerebral depressant and hypnotic effects (3). The spectral analysis of the electroencephalogram (EEG) may provide specific information with regard to the status of the brain. For example, the slow wave may reflect the homeostatic processes of sleep (4). δ (0.5–4 Hz) EEG power is highest at the start of the night when the need for recuperation is greatest and decreases during the night as this requirement is met. However, the current case report presents a female patient whose EEG recording demonstrated a rare generalized 3 Hz δ frequency band in the daytime during waking status. To the best of our knowledge, this study is the first report prolonged menstruation with an abnormal δ EEG power.

## Case report

A healthy 51-year-old female with no history of head trauma, coffee or alcohol consumption and contraceptive drug use was admitted to the Department of Urology (Second Xiangya Hospital, Central South University, Changsha, China). The patient had not previously experienced postpartum headaches, but had suffered from heart palpitations and dizziness for 6 months and was receiving treatment in the Department of Neurology. The study was approved by the ethics committee of Central South University, Changsha, China. Written informed consent was obtained from the patient.

**Figure 1:**
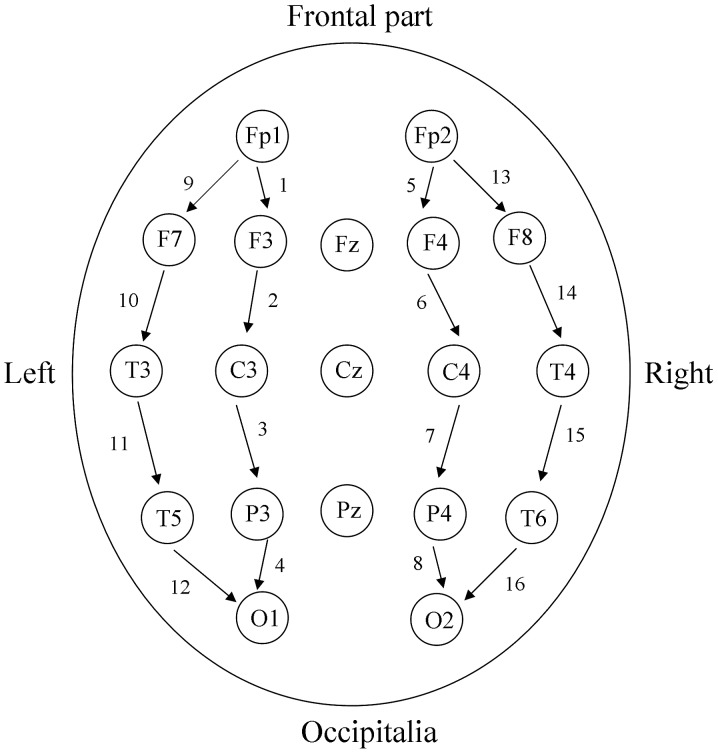
Placement of 32 electrodes for electroencephalogram with 16-channel longitudinal bipolar montage.

**Figure 2:**
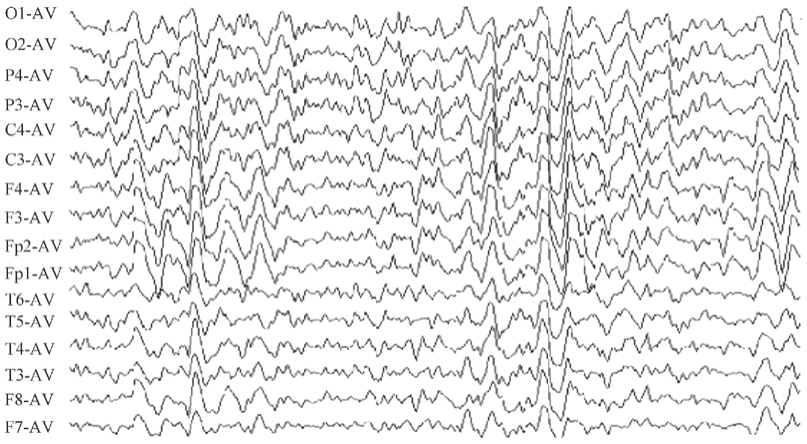
Electroencephalogram spectrum of the patient with generalized δ frequency in 16 channels.

The patient had experienced prolonged menstruation and increased menstrual blood volume for 6 years. Due to dizziness and palpitations, the patient was admitted to the Department of Urology and further evaluations were performed. General physical and neurological examination results were normal. The routine laboratory tests showed mild anemia of the patient. Gynecological examination revealed secondary anemia and hysteromyoma. Upon admission, hemoglobin levels were low at 69 g/l; however, levels increased to 115 g/l following blood transfusion and resection of the hysteromyoma. EEG activity was continuously recorded at rest with 32 electrodes using the standard EEG electrode placement (Fig. 1). The EEG examination results indicated a generalized 3 Hz δ frequency band with 30–80 μV power and a long-range δ frequency band during hyperventilation (Fig. 2). Computed tomography and magnetic resonance imaging scans of the head of the patient showed no significant abnormalities. There was no significant improvement in the EEG recordings one month following the first EEG examination.

## Discussion

The current case report presents a female with prolonged menstruation and increased menstrual blood volume whose EEG recording demonstrated a rare generalized 3 Hz δ frequency band in the waking state. A previous epidemiological study of generalized δ frequency showed that a 3 Hz δ frequency band mainly occurs following brain injury, such as encephalitis or mental confusion, and during the sleeping state (5). Although a small number of individuals exhibit a slightly abnormal δ frequency in EEG, the observations in the present case report are extremely rare. Furthermore, the patient demonstrated a unique and generalized δ frequency with no cerebral injury or brain disease. This indicated an obvious abnormality of cerebral activity.

EEG δ frequency is enhanced during non-rapid eye movement sleep following sleep deprivation and therefore, EEG δ frequency is used as a parameter to model process S (homeostatic) in the two-process model of sleep (6). Previously, several studies have indicated that EEG δ frequency is regulated independently of sleep duration (7,8). For example, high amplitude EEG δ frequency occurs during the waking state following systemic atropine administration or hyperventilation in children (9,10). Several sleep regulatory substances may enhance the EEG δ frequency, such as tumor necrosis factor-α, growth hormone releasing hormone and interleukin-1, all of which may induce a change in the duration of sleep (11).

In the present case report, generalized δ frequency was found to correlate with estrogen levels in a patient with prolonged menstruation. According to previous studies, a general increase in brain activation may be associated with enhanced estradiol levels, such as EEG powers (12). However, to the best of our knowledge, this case study was the first to demonstrate enhanced δ frequency levels under the specific conditions of the patient. Therefore, we hypothesize that the prolonged menstruation and increased menstrual blood volume may have enhanced estrogen levels and triggered the generalized δ frequency.

In conclusion, the present report evaluates an unusual case involving a female patient who had prolonged menstruation and an abnormal δ EEG power. The results illustrated that the prolonged menstruation and increased menstrual blood volume may have induced the generalized δ frequency without brain injury.
